# Supplementary material for: Using an ordinary differential equation model to separate rest and task signals in fMRI
Source: Nat Commun. 2025 Aug 3;16:7128. doi: 10.1038/s41467-025-62491-6 (PMC12317992; doi:10.1038/s41467-025-62491-6)
Supplement: Supplementary file 2 — Reporting Summary [file 41467_2025_62491_MOESM2_ESM.pdf]

nature portfolio

Corresponding author(s): Amrit Kashyap

Last updated by author(s): 2024.8.14

# Reporting Summary

Nature Portfolio wishes to improve the reproducibility of the work that we publish. This form provides structure for consistency and transparency in reporting. For further information on Nature Portfolio policies, see our Editorial Policies and the Editorial Policy Checklist.

## Statistics

For all statistical analyses, confirm that the following items are present in the figure legend, table legend, main text, or Methods section.

| n/a | Confirmed | |
|---|---|---|
| ☐ | ☒ | The exact sample size (*n*) for each experimental group/condition, given as a discrete number and unit of measurement |
| ☐ | ☒ | A statement on whether measurements were taken from distinct samples or whether the same sample was measured repeatedly |
| ☐ | ☒ | The statistical test(s) used AND whether they are one- or two-sided<br>*Only common tests should be described solely by name; describe more complex techniques in the Methods section.* |
| ☐ | ☒ | A description of all covariates tested |
| ☐ | ☒ | A description of any assumptions or corrections, such as tests of normality and adjustment for multiple comparisons |
| ☐ | ☒ | A full description of the statistical parameters including central tendency (e.g. means) or other basic estimates (e.g. regression coefficient) AND variation (e.g. standard deviation) or associated estimates of uncertainty (e.g. confidence intervals) |
| ☐ | ☒ | For null hypothesis testing, the test statistic (e.g. *F*, *t*, *r*) with confidence intervals, effect sizes, degrees of freedom and *P* value noted<br>*Give P values as exact values whenever suitable.* |
| ☒ | ☐ | For Bayesian analysis, information on the choice of priors and Markov chain Monte Carlo settings |
| ☒ | ☐ | For hierarchical and complex designs, identification of the appropriate level for tests and full reporting of outcomes |
| ☒ | ☐ | Estimates of effect sizes (e.g. Cohen's *d*, Pearson's *r*), indicating how they were calculated |

*Our web collection on statistics for biologists contains articles on many of the points above.*

## Software and code

Policy information about availability of computer code

| | |
|---|---|
| Data collection | Open Source from the Human Connectome Project |
| Data analysis | Custom code in python available at https://github.com/sagastra/fMRI_SiNDy |

For manuscripts utilizing custom algorithms or software that are central to the research but not yet described in published literature, software must be made available to editors and reviewers. We strongly encourage code deposition in a community repository (e.g. GitHub). See the Nature Portfolio guidelines for submitting code & software for further information.

## Data

Policy information about availability of data

All manuscripts must include a data availability statement. This statement should provide the following information, where applicable:

- Accession codes, unique identifiers, or web links for publicly available datasets
- A description of any restrictions on data availability
- For clinical datasets or third party data, please ensure that the statement adheres to our policy

Human Connectome Project doesnt allow to share processed data. We have included how we processed the data from the open source database.

## Research involving human participants, their data, or biological material

Policy information about studies with human participants or human data. See also policy information about sex, gender (identity/presentation), and sexual orientation and race, ethnicity and racism.

| | |
|---|---|
| Reporting on sex and gender | No reports on Sex and Gender |
| Reporting on race, ethnicity, or other socially relevant groupings | No reports on Race, Ethnicity or other socially relevant groupings |
| Population characteristics | The population is from the HCP healthy young adults cohort aged 22-35 matched with ethnicity and gender. |
| Recruitment | Participants were recruited in different locations since this was a multisite study. There is a bias to the large inclusion of twin data which was addressed in the analysis by training a model with only twins and non twins and showing no difference in results. |
| Ethics oversight | Approved by Charite Medizin University Ethics board for the use of HCP open access and restricted data. |

Note that full information on the approval of the study protocol must also be provided in the manuscript.

# Field-specific reporting

Please select the one below that is the best fit for your research. If you are not sure, read the appropriate sections before making your selection.

☒ Life sciences ☐ Behavioural & social sciences ☐ Ecological, evolutionary & environmental sciences

For a reference copy of the document with all sections, see nature.com/documents/nr-reporting-summary-flat.pdf

# Life sciences study design

All studies must disclose on these points even when the disclosure is negative.

| | |
|---|---|
| Sample size | Sample size was determined by how much data was needed to train the SINDy algorithm. It required about half a million timepoints which required the entire subject list for most HCP Tasks. For the Rest data we utilized the smaller Q1 release of 447 participants using all four recordings. Since the Task data is much shorter and had only two recordings per individual, we downloaded as much Task data that was available, and downloaded 1032 subjects for Working Memory, 1013 for Relational, 1017 for Emotion, and 1032 for Gambling. |
| Data exclusions | Data from subjects that did not have two Task runs |
| Replication | The ODEs for Rest and Task converged to a set of coefficients once the sample size was large enough which we tested using randomized subsets of the population. We replicated the behavioral results on 4 HCP tasks as well as self contained 14 subtask catogories. We also replicated our results by using twin participants only and non-twin participants only and showed no difference exists between the two groups. For the algorithms employed such as the Elastic Net and SVM, we used 10 fold cross validation to ensure replicability. |
| Randomization | The randomization was performed in two ways: 1) using a 80/20 split of fMRI files in all the appropriate areas of the analysis. This includes the results for the model (Figure 3) as well as for the subsequent behavioral tests 2) radomizing within the twin group and non-twin group participants (also using a 80/20 split). Moreover since HCP had multiple fMRI files per subject, we made sure that the same subjects files were randomized together in the test training split. |
| Blinding | There were no groups in our study design, and the whole population was used to train a single set of equations to describe the brain dynamics. The only 'groups' that were considered, were the different separation strategies, and no blinding was perfomed as they were all constructed by applying the ODEs to predict and remove task independent signals. |

# Reporting for specific materials, systems and methods

We require information from authors about some types of materials, experimental systems and methods used in many studies. Here, indicate whether each material, system or method listed is relevant to your study. If you are not sure if a list item applies to your research, read the appropriate section before selecting a response.

## Materials & experimental systems

| n/a | Involved in the study |
|---|---|
| x | Antibodies |
| x | Eukaryotic cell lines |
| x | Palaeontology and archaeology |
| x | Animals and other organisms |
| x | Clinical data |
| x | Dual use research of concern |
| x | Plants |

## Methods

| n/a | Involved in the study |
|---|---|
| x | ChIP-seq |
| x | Flow cytometry |
| | x MRI-based neuroimaging |

# Plants

Seed stocks: NA

Novel plant genotypes: NA

Authentication: NA

# Magnetic resonance imaging

## Experimental design

Design type: Rest and Tasks (Gambling, Working Memory, Relational and Emotion) block design but anaylsis performed on each trial

Design specifications: The block and event design varied from Task to Task. We analyzed them based on event based trials each that were embedded in different block designs (some having multiple subtasks within a block and some having separate subtasks per block). The temporal spacing between each event and the blocks were different in all trials, but these did not effect the results as they generalized aross the tasks no matter the design specification.

Behavioral performance measures: The behavioral data attached to the Task data were extracted from the behavioral tables. For the Working Memory Task these include the RT, the difficulty of the Task 0 vs. 2 back, the type of Task (Face, Place, Body, Tools), and the e-prime timings relative to the fMRI scan for each of the 80 trials in each WM scan. For the Gambling Task these included RT, Punish or Reward trial, and the e-prime timings relative to the fMRI scan for each of the 32 trials in each Gambling scan. For the Relational Task these included RT, Shape or Texture trial, and the e-prime timings relative to the fMRI scan for each of the 27 trials in each Relational scan. For the Emotional Task these included RT, Fear or Neutral trial, and the e-prime timings relative to the fMRI scan for each of the 30 trials in each Emotional scan. Since the Emotional script had a known bug during HCP recording, where only half of the last trial was recorded, we chose to truncate the last task block and thus only had 30 trials per scan.

## Acquisition

Imaging type(s): functional, structural, diffusion

Field strength: 3T

Sequence & imaging parameters: Parallel Pulse EPI for the fMRI scans. More detail on HCP documents

Area of acquisition: whole brain including subcortical areas

Diffusion MRI: [x] Used [ ] Not used

Parameters: Tracktography was perfomed using a well validated pipeline for HCP data based on Mrtrix published by another group Civier 2019. In short, they use the bvals and bvecs from HCP to denoice and then perform ACT to determine the fibers.

## Preprocessing

Preprocessing software: Data downloaded was already preprocessed using HCP minimal processing pipeline and then transformed into T1 native space using FSL.

Normalization: The data was averaged, filtered and z-transformed based on the ROI boundaries.

| | |
|---|---|
| Normalization template | We used the T1 native space to create atlas boundaries of the Desikan Killiany in high resolution (0.75 mm). We then transformed the fMRI signal to native T1 space and averaged all voxels for each given boundary. |
| Noise and artifact removal | We filtered the signal from a 0.008-0.1 range to minimize the effect of breathing and heartrate. No ICA denoising was performed as it has been shown that it reduces behavioral correlates in Task fMRI. Also other strategies such as PCA or other denoising introduced strange effects on SINDy coefficients. Instead, we utilized SINDy's own in built packages that denoise the signal in the derivative space and improves the convergence of the coefficients. This worked better for our approach of fitting ODEs using SINDy to describe the fMRI data. SINDY can also censor a percentage of samples, but this had no effect in the coeffcients with sufficiently large datasets. |
| Volume censoring | Using the SINDy software we used the smoothed finite difference function to calculate the derivative that would address outliers in the timeseries. SINDY can also censor a percentage of samples, but this had no effect in the coeffcients with sufficiently large datasets. |

## Statistical modeling & inference

| | |
|---|---|
| Model type and settings | The prediction of the fitted ODE Rest and Task dynamics are used to remove the task independent components of the signal based on the different separation strategies (see Effect(s) below). The signal correlation to reaction time is performed directly on the spatial temporal signal as well as integrated using an Elastic Net to predict a single reaction time. The distribution of correlation and r-squared of the different separation strategy groups are then tested to determine which separation strategy is significant using a one tailed t-test. Additionally we simulated a null model signal by matching the variance and mean of the signal to show that this random signal only reduces associations with behavioral variables. |
| Effect(s) tested | The focus of the study was to test different separation strategies that would describe the Rest and Task dynamics. We tested three models primarily Active Cortex, Rest Baseline, and Task Baseline Model, as well as a generalized form resulting from a linear combination of these strategies to determine the relationship between Rest and Task. We tested in two ways: in the strategies ability to improve relations to the reaction time performed at each trial and how the functional connectivity of the respective Tasks diverge in high dimentional space in order to confirm that we have reduced the Task independent component of the signal. |

Specify type of analysis: ☐ Whole brain ☒ ROI-based ☐ Both

| | |
|---|---|
| Anatomical location(s) | The Desikan Killiany atlas was used as the parcellation scheme which contains 68 cortical areas and 16 subcortical areas for a total of 84 regions to represent the central nervous system. Since we used a generalized approach for any Task there was no need to exclude any ROIs. |
| Statistic type for inference (See Eklund et al. 2016) | NA, we identify brain regions purely on there correlation with reaction time (no GLM model) but show the regions with largest correlation correspond to areas of previous HCP analysis of the data using voxel based analysis. |
| Correction | Each of the afermonetioned strategies (see Effect(s) tested) were tested on how they changed the relationship to behavioral measures on each of the 14 subtasks separately, but since these are not many tests we did not use correction. The Elastic Net analysis also showed for the Active Cortex Model a mean increase in r-squared by 9 percent across the 14 subtasks which is significant to at least 3-5 sigmas. |

## Models & analysis

| n/a | Involved in the study |
|---|---|
| ☐ | ☒ Functional and/or effective connectivity |
| ☐ | ☒ Graph analysis |
| ☐ | ☒ Multivariate modeling or predictive analysis |

| | |
|---|---|
| Functional and/or effective connectivity | Effective connectivity is calculated fitting a Taylor polynomial between each pair of edges, and is one of the novel methods introduced in this manuscript. Functional Connectivity is calculated to evaluate one component of the ODE model and is calculated using Pearson Correlation. |
| Graph analysis | We use a Network ODE model similar to those of older Brain Network Model, but is constructed via data driven approaches using constraints on the hemodynamic response function. The graph is constructed by fitting a polynomial edge between each pair of ROIs. There are no symmetry constraints, although the solutions that SINDy yields end up being very close to symmetrical. The ODEs are constructed for group level. |
| Multivariate modeling and predictive analysis | The independent variables were the timeseries taken after 15 sec after the stimulus onset. These were matched to the reaction time using Pearsons correlation at each spatial temporal point as well as combined using an Elastic Net to predict a singular reaction time. For the UMAP, we used a SVM to separate out the different catogeries using the two UMAP components. We tested both the SVM and the Elastic Net using a 10 fold Cross Validation appraoch. |
